# Eco-Friendly Approach to Produce Durable Multifunctional Cotton Fibres Using TiO_2_, ZnO and Ag NPs

**DOI:** 10.3390/nano12183140

**Published:** 2022-09-10

**Authors:** Monika Ivanuša, Blažka Kumer, Elizabeta Petrovčič, Danaja Štular, Matija Zorc, Ivan Jerman, Marija Gorjanc, Brigita Tomšič, Barbara Simončič

**Affiliations:** 1 Faculty of Natural Sciences and Engineering, University of Ljubljana, Aškerčeva 12, 1000 Ljubljana, Slovenia; 2 National Institute of Chemistry, Hajdrihova 19, 1000 Ljubljana, Slovenia

**Keywords:** cotton, TiO_2_, ZnO, Ag, nanocomposite coating, antimicrobial, UV protection, multifunctionality, durability

## Abstract

The development of durable multifunctional properties is crucial for the production of high-performance technical textiles. In this work, a novel, environmentally friendly and facile method was developed for the chemical modification of cotton fabric by in situ biosynthesis of Ag NPs in the presence of sumac leaf extract as a reducing agent on TiO_2_, ZnO and TiO_2_ + ZnO previously applied to cotton fibres. The results showed that the presence of TiO_2_, ZnO and TiO_2_ + ZnO significantly increased the concentrations of the synthesised Ag NPs on the cotton fibres compared to the one-component Ag coating. This resulted in excellent antimicrobial properties of the TiO_2_/Ag, ZnO/Ag and TiO_2_ + ZnO/Ag composites even after 25 washes. While the TiO_2_ and ZnO particles in the composite were incompatible, the synergistic effect among Ag, TiO_2_ and ZnO in the composites resulted in excellent UV blocking properties of the coatings before and after 25 washes. Since the biosynthesis of Ag NPs was accompanied by a yellow–brown colouration of the samples, the photocatalytic self-cleaning of the composite coating could not be determined from the photodegradation rate of the coffee stains. This research provides a new environmentally friendly approach to producing durable antimicrobial and UV blocking coatings on cotton fibres.

## 1. Introduction

Nanotechnology, driven by green chemistry, has become the main eco-friendly technology for the chemical and morphological modification of textile fibres to produce high-performance, value-added technical textile products. It prescribes the synthesis and application of nanomaterials on textile substrates considering the principles of green chemistry to avoid hazardous chemicals via green alternatives [1]. Among the nanomaterials, inorganic nanoparticles (NPs) and nano/microstructures have emerged as promising chemical finishing agents to create nanocomposite textile substrates with multiple protective properties, such as photocatalytic self-cleaning, antimicrobial activity and UV blocking properties, as well as hydrophobicity, electrical conductivity and thermal stability [2].

Silver (Ag), TiO_2_ and ZnO in the form of NPs represent some of the most important and widely used inorganic nanomaterials for the chemical modification of textiles due to their exceptional physicochemical properties [3,4,5]. Ag NPs, as representatives of noble metal nanomaterials, show excellent antimicrobial activity against a wide range of bacteria, viruses, fungi, moulds, yeasts and algae, even at very low concentrations, which makes them one of the leading players in the production of medical textiles and healthcare materials [6,7]. TiO_2_ and ZnO are transition metal oxides known as n-type wide-bandgap semiconductors and have bandgap energies of 3.0–3.2 and 3.2–3.4 eV, respectively, which enables them to have photocatalytic activity under UV light [8,9]. For textile applications, TiO_2_ and ZnO NPs have been recognized as effective photocatalytic, self-cleaning, UV blocking and antimicrobial agents. Both semiconductors also exhibit biocompatibility, non-toxicity and recyclability, which are of great importance from an environmental point of view [10,11]. 

In order to enhance the photocatalytic activity of TiO_2_ and ZnO NPs and shift this activity to the visible light region, various surface and interface modifications strategies of TiO_2_ and ZnO have been developed, among which doping/loading of metals acting as plasmonic absorbers and current collectors has been found to be very effective. In several studies, nanocomposites of Ag/TiO_2_ [12,13,14,15,16,17,18] and Ag/ZnO [19,20,21,22] were successfully synthesized and applied to textile fibres to improve their photocatalytic self-cleaning and antimicrobial activities and UV blocking properties compared to fibres coated with only TiO_2_ or ZnO NPs. The main contribution to the enhanced photocatalytic activity of the Ag/TiO_2_ and Ag/ZnO nanocomposites is considered to be the transfer of photogenerated electrons from the semiconductors to the Ag NPs, which then reduce molecular oxygen to superoxide radicals that can participate in the photochemical degradation of various pollutants. To follow the green chemistry approaches, some studies have demonstrated the biosynthesis of Ag NPs using plant extracts as reducing and stabilizing agents [16] or performed silver photoreduction under UV light irradiation without adding chemical agents [15].

Another strategy to enhance the photocatalytic activity of TiO_2_ and ZnO NPs is the creation of TiO_2_–ZnO heterojunctions [23,24,25,26,27,28,29,30,31,32], where the transfer of photogenerated electrons and holes between the two semiconductors increases the charge separation and decreases the electron–hole recombination rate [23]. It was found that the photodegradation efficiency of nanofibers with the incorporation of TiO_2_–ZnO nanocomposites was significantly improved compared to that of the ZnO- and/or TiO_2_-incorporating nanofibers due to the enhanced separation efficiency of charge carriers resulting from the charge transfer between ZnO and TiO_2_ [25,27]. When TiO_2_–ZnO nanorods were applied to textile fibres, the antimicrobial activity and photodegradation ability of the fibres were significantly increased compared to fibres treated with ZnO alone [30,32]. Another study reported that multiple durable properties through synergistic effects of TiO_2_ and ZnO in a hybrid TiO_2_–ZnO nanocrystal composite were achieved on textile fibres, including exceptional antimicrobial activity, protection against UV radiation and photooxidative degradation of stains [31]. 

In recent years, heterostructure nanocomposites, including Ag, TiO_2_ and ZnO, have already been synthesized and used for water purification and antimicrobial performance [33,34,35,36,37,38,39,40]. It was found that the three-component Ag/TiO_2_/ZnO nanocomposites synthesized in various ways had much higher photocatalytic performance in the degradation of Methylene blue, Rhodamine B, Reactive black 5, Malachite green, tetracycline hydrochloride and formic acid as model pollutants under UV and visible light irradiation compared to a TiO_2_/ZnO nanocomposite or TiO_2_ and ZnO alone [33,34,35,37,38,40]. Its antimicrobial activity was also confirmed [33,35,37]. In contrast, another study demonstrated that Ag doping of a TiO_2_–ZnO nanocomposite negatively affected its photocatalytic efficiency in the degradation of Methylene blue, Rhodamine B and Malachite green in aqueous solutions under visible light [39]. The Ag/TiO_2_/ZnO nanocomposite has also been incorporated into nanofibers used in dye-sensitized solar cells [36]. 

Thus far, to our knowledge, there are no reports in the literature dealing with the synthesis of Ag/TiO_2_/ZnO nanocomposites in the presence of textile fibres to obtain a durable multifunctional coating, which is still a challenging research topic. This encouraged us to develop a novel, facile and environmentally friendly method for the chemical modification of cotton fibres, in which a two-component sol-containing TiO_2_ and ZnO NPs are applied to the fibres in a first step, followed by the in-situ biosynthesis of Ag NPs in the presence of sumac leaf extract as a reducing agent and the textile substrate as the stabilizing agent. The selection of the sumac leaf extract as a green reducing agent was not by chance. Indeed, the sumac plant (*Rhus typhina* L.), native to North America, is used in soil conservation, sand stabilization and urban reforestation in many countries. Since it is considered as an invasive plant species in non-native habitats, its collection and use benefit the environment. Moreover, the sumac leaf extract is rich in phenolic compounds, mainly of gallic acid (3,4,5-trihydroxybenzoic acid), which is well known as a very effective reducing agent in the synthesis of Ag NPs. Accordingly, the results of our previous studies showed that face-centred cubic Ag NPs with an average size of 52 to 105 nm could be generated on the cotton fibres in the reduction of an AgNO_3_ precursor using the phenolic compounds of sumac leaf extract as reducing agents [41]. For comparison, two-component TiO_2_/Ag and ZnO/Ag and one-component TiO_2_, ZnO and Ag coatings were applied under the same conditions as the three-component coating. The chemically modified cotton fibres were characterized by SEM, EDS and ICP-MS analyses. The antimicrobial activity, UV blocking and photocatalytic self-cleaning efficiency of the coatings were evaluated, focusing on the washing resistance of the functions.

## 2. Materials and Methods

### 2.1. Materials

Alkali-scoured, bleached and mercerized 100% cotton fabric weighing 120 g/m^2^ (warp density: 51 threads/cm; weft density: 31 threads/cm) was purchased from Tekstina d.d., Slovenia. Commercial titanium (IV) oxide, anatase nano powder with particles less than 25 nm in size and ZnO nano powder with 30 nm particles and silver nitrate (AgNO_3_) were purchased from Sigma Aldrich. The Rhodamine B (Rh B) dye was purchased from Sigma Aldrich and used without further purification.

The reactive organic–inorganic sol–gel precursor iSys HPX (CTH Gruppe, Tübingen, Germany) was used. Sumac leaf extract was prepared according to the literature [42]. Deionized water was used throughout the study. 

### 2.2. Fabrication of CO/TiO_2_/Ag, CO/ZnO/Ag and CO/TiO_2_ + ZnO/Ag Samples

To increase the adsorption of TiO_2_, ZnO and AgNO_3_, the cotton fabric (sample code CO) was pretreated with a polysiloxane matrix. To this end, the reactive organic–inorganic sol–gel precursor iSys HPX was prepared in distilled water at a concentration of 15 g/L and applied to cotton fabric by the pad–dry–cure method with 80 ± 2% wet pick-up, drying at 100 °C for 1 min and curing at 150 °C for 4 min. The samples were then stored at 65 ± 2% relative humidity and 20 ± 2 °C for 7 days to allow the siloxane matrix to fully form. 

To fabricate CO/TiO_2_ and CO/ZnO samples, one-component TiO_2_ and ZnO sols were prepared at a concentration of 3% in distilled water and sonicated for 2 h to obtain homogeneous sols. Then, the sols were applied to the cotton fabric samples using the pad–dry–cure method with 95 ± 2% wet pick-up, dried at 100 °C for 1 min and cured at 150 °C for 5 min. For the fabrication of CO/TiO_2_ + ZnO, a concentration of 1.5% for each component was used to prepare the two-component TiO_2_ + ZnO sol. It was applied to the cotton fabric samples under the same conditions as the one-component sols. 

**Scheme 1:**
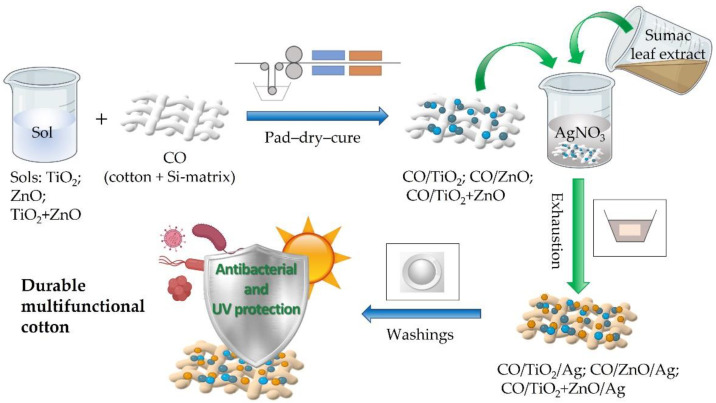
The preparation of durable multifunctional cotton samples.

To obtain CO/TiO_2_/Ag, CO/ZnO/Ag and CO/TiO_2_ + ZnO/Ag samples, Ag NPs were in situ synthesized into CO/TiO_2_, CO/ZnO and CO/TiO_2_ + ZnO samples. In the synthesis procedure, cotton samples were immersed in a 0.5 mM solution of AgNO_3_ at a 25:1 liquor ratio. The samples were stirred in the AgNO_3_ solution in a Gyrowash apparatus (James Heal, Halifax, UK) for 10 min at 60 °C, which allowed the Ag^+^ ions to be well dispersed and adsorbed onto the cotton fibres. The solution of the sumac extract was then added to the samples to obtain a final 50:1 liquor ratio. The samples were shaken for an additional 60 min at 60 °C in the Gyrowash, rinsed with distilled water and air dried at room temperature. For comparison, the in-situ synthesis of Ag NPs was performed on the cotton sample pretreated with the polysiloxane matrix without the presence of TiO_2_ and ZnO to obtain CO/Ag sample. The preparation of durable multifunctional cotton samples is shown in Scheme 1.

### 2.3. Washing 

The chemically modified cotton samples were washed in a Gyrowash 815 (James Heal, UK) test apparatus according to the EN ISO 105 C06 standard. Washing was performed in a solution of 4 g/L ECE phosphate reference detergent B at a 50:1 liquor ratio at 40 °C for 45 min in the presence of ten steel balls, providing an accelerated wash treatment equivalent to five household washes. After washing, the samples were rinsed in distilled water at 40 °C, rinsed in cold tap water and dried at room temperature. 

### 2.4. Analysis and Measurement

#### 2.4.1. Scanning Electron Microscopy (SEM) and Energy-Dispersive X-ray Spectroscopy (EDS)

SEM images of the untreated and chemically modified cotton samples were acquired using a JSM 6060 LV scanning electron microscope (JEOL, Tokyo, Japan) operated with a primary electron beam accelerated to 10 kV. All samples were coated with a thin layer of gold before examination to provide conductivity and improve the quality of the images.

EDS analysis was performed using a field emission scanning electron microscope, FEG-SEM Thermo Scientific Quattro S (ThermoFischer Scientific, Waltham, MA, USA). Sample analysis was performed using an Oxford Instruments Ultim Max 65 energy-dispersive detector (EDS) and AZtec software Ver 6.0 (Oxford Instruments, Santa Barbara, CA, USA). Samples were coated with a thin carbon layer prior to analysis to provide conductivity and thus improve the quality of the images.

#### 2.4.2. Fourier Transform-Infrared (FT-IR) Spectroscopy

Infrared spectra of the untreated and chemically modified cotton samples were performed using a Vertex 70 V (Bruker, Berlin, Germany) integrated with an attenuated total reflection (ATR) accessory (Bruker, Germany) with a diamond crystal (*n* = 2.0). Spectra were recorded over the range 4000–300 cm^−^^1^ with a resolution of 4 cm^−1^ and an average set of 128 spectra per sample.

#### 2.4.3. X-ray Diffraction (XRD)

XRD characterization of the untreated and chemically modified cotton samples was performed using a PANalyticalX’Pert PRO X-ray diffractometer (XRD) (CuK ∝ 1 = 1.5406 Å) with a fully open X’Celerator detector (2.122° 2θ). The XRD pattern was measured from 10 to 80° 2θ with a step size of 0.034° 2θ and 100 s integration time.

#### 2.4.4. Inductively Coupled Plasma-Mass Spectroscopy (ICP-MS)

The concentrations of Ag, Ti and Zn in the chemically modified samples were determined by ICP-MS using a Perkin Elmer SCIED Elan DRC spectrophotometer. A sample of 0.5 g was prepared in a Milestone microwave system by acid decomposition with 65% HNO_3_ and 30% H_2_O_2_. Concentrations were reported as the mean values of two measurements for each sample. Based on the measured values of Ti and Zn, the concentrations of TiO_2_ and ZnO were calculated. 

#### 2.4.5. UV–Vis Spectroscopy

Transmission spectra of untreated and chemically modified cotton samples were recorded using a Lamda 850+ UV–Vis spectrophotometer (Perkin Elmer, Buckinghamshire, United Kingdom) equipped with a reflection module—a 150 mm integration sphere—and fully controlled by a computer with UV software WinLab Ver 6.0. Transmittance (T) was measured in the wavelength range of 250–750 nm. Three measurements were made of each sample at different angles of warp alignment, and the average value of T at each wavelength was calculated. The transmission spectra were converted to absorption spectra using the following equation:

(1) A=−logT,

where A is absorbance. 

#### 2.4.6. Antibacterial Activity

Bacterial reduction by the chemically modified samples was evaluated against the Gram-negative bacteria *Escherichia coli* (*E. coli*; ATCC 25922) using the ASTM E2149-01 standard method. A fabric sample of 1 g was immersed in 20 mL of a bacterial suspension of 105 CFU/mL in a flask, which was then shaken for 1 h with a wrist-action shaker. Subsequently, 40 μL of each suspension was spread on nutrient agar and incubated for 24 h at 37 °C. Two parallels were performed for each sample, and each parallel was distributed on 4 agar plates, resulting in a total of eight counts per sample. The reduction in bacterial growth, R, was calculated as follows [43]:

(2) $$R=\frac{B-A}{B}\;\cdot \;100\;\left(\%\right),$$

where B is the number of bacterial-colony-forming units (CFU) recovered from the inoculated untreated control sample in the flask at an incubation time of 24 h, and A is the number of bacteria recovered from the inoculated functionalized test sample in the flask after an incubation time of 24 h.

#### 2.4.7. UV Protection Properties 

The UV protective properties of the untreated and chemically modified fabric samples were determined by measuring the UV transmission through the samples according to the EN 13758-1:2002 Standard using a Lambda 850+ UV–Vis spectrophotometer (Perkin Elmer, United Kingdom) equipped with a reflectance module with a 150 mm integration sphere and fully controlled by a computer running UV software WinLab Ver 6.0. Six measurements were made of each sample at different angles of warp alignment. The T values were determined in the wavelength range of 280–400 nm, and the arithmetic mean of T was calculated at wavelengths of 315–400 nm (T (UVA)), 290–315 (T (UVB)) and 290–400 (T (UVR). The UV protection factor (UPF) was calculated using the following equation [44]:

(3) $$\mathrm{UPF}=\frac{\sum_{290}^{400}E\left(\lambda \right)\;\varepsilon \left(\lambda \right)\;\Delta \left(\lambda \right)}{\sum_{290}^{400}E\left(\lambda \right)\;\varepsilon \left(\lambda \right)\;T\left(\lambda \right)\;\Delta \left(\lambda \right)},$$

where E(λ) is solar spectral irradiance, ɛ(λ) is relative erythemal effectiveness, Δ(λ) is the wavelength interval and T(λ) is the spectral transmittance at wavelength λ. UPF rating and protection categories were determined from the UPF values calculated according to the Australian/New Zealand Standard for Sun Protective Clothing—Evaluation and Classification (AS/NZS 4399, 2017) [45], where UPF values of 15–24 correspond to UPF ratings of 15 and 20 and a protection category of “good”; UPF values of 25–39 correspond to UPF ratings of 25, 30 and 35 and a protection category of “very good” and UPF values of 40–50 and above correspond to UPF ratings of 40, 45, 50, 50+ and a protection category of “excellent.”

#### 2.4.8. Photocatalytic Activity

The photocatalytic degradation of Rh B dye was studied in the presence of the untreated and chemically modified samples. The studied samples were placed in a cuvette filled with 3 mL of 0.025 mM Rh B solution and illuminated in a Xenotest Alpha instrument (Atlas, Mount Prospect, IL, USA) equipped with a visible xenon arc lamp (radiation attitude 0.8–2.5 kVA and extended radiation range 300–400 nm). The cuvettes were illuminated for 30, 60, 120, 160 and 240 min. After each illumination time, the absorbance of the Rh B solution was measured at λ_max_ and the corresponding concentration of Rh B dye was determined using a previously prepared calibration curve. The measurements were performed using a Lamda 850+ UV–Vis spectrophotometer (Perkin Elmer, United Kingdom). The degree of Rh B degradation was determined as the ratio c_t_/c_0_, where c_t_ is the concentration of Rh B solution after the illumination time and c_0_ is the initial concentration of Rh B solution after the adsorption–desorption equilibrium was established. The higher the degree of Rh B degradation, the lower the c_t_/c_0_ ratio.

The photocatalytic self-cleaning activity of the untreated and chemically modified samples was determined based on the photodegradation of a coffee stain under simulated sunlight. For this purpose, the samples were immersed in decanted Turkish coffee (5 g ground coffee/100 mL water) for 30 s, then air dried and illuminated in a Xenon Alpha instrument (Atlas, USA) at 35 °C and 70% humidity for eight hours. Before and after each hour of illumination, the colour coordinates L*, a* and b* in CIELAB colour space were determined for the studied samples using a Datacolor Spectro 1050 spectrophotometer (Datacolor, Lawrenceville, NJ, USA). Measurements were performed with a 9 mm aperture under D65 illumination and an observation angle of 10°. Ten measurements were performed for each sample, and the colour difference (Δ$E_{ab}^{*}$) was calculated using the following equation [46]:

(4) $$\Delta E_{ab}^{*}=\sqrt{{\left(\Delta L^{*}\right)}^{2}+{\left(\Delta a^{*}\right)}^{2}+{\left(\Delta b^{*}\right)}^{2}},$$

where ΔL*, Δa* and Δb* are the differences between the lightness, green–red and blue–yellow colour coordinates, respectively, calculated between the illuminated and non-illuminated samples.

The test of photo-degradation of coffee stains on the studied samples was performed under the same conditions, and the digital images of the samples were taken before and after eight hours of illumination to compare the changes in the colour of the coffee stains.

## 3. Results and Discussion

### 3.1. Morphological, Chemical and Optical Properties 

**Figure 1:**
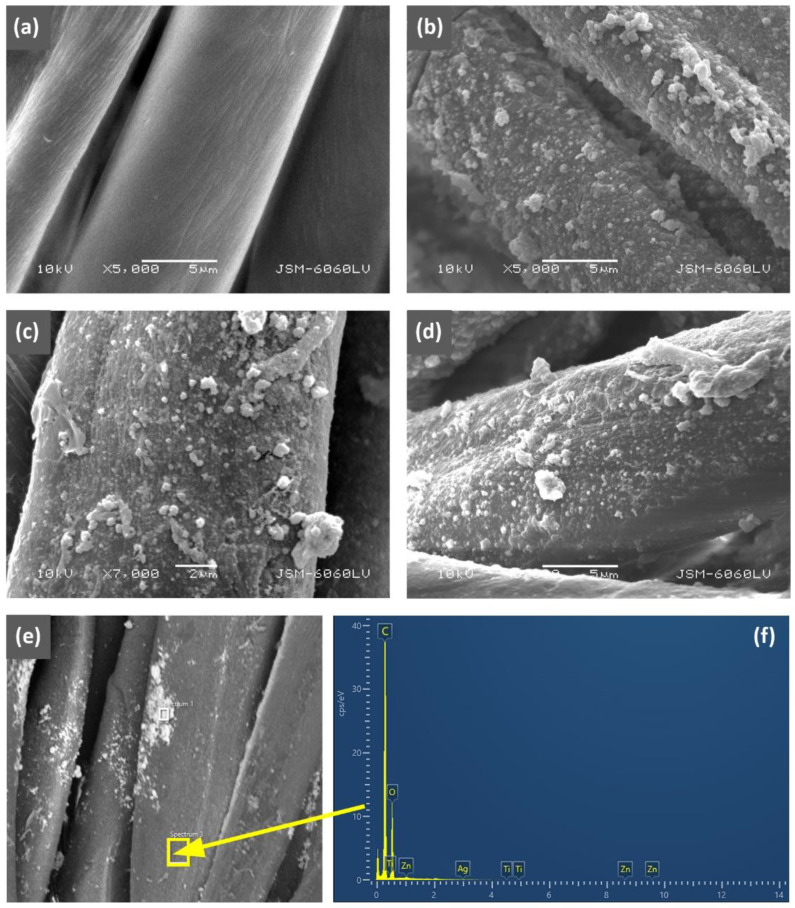
SEM images of untreated (**a**) and chemically modified cotton samples: CO/TiO_2_/Ag (**b**), CO/ZnO/Ag (**c**) and CO/TiO_2_ + ZnO/Ag (**d**). SEM/BSE image of CO/TiO_2_ + ZnO/Ag sample (**e**). EDS spectrum acquired from CO/TiO_2_ + ZnO/Ag sample in place bordered with yellow (**f**).

The morphological and structural changes in the cotton fibres due to the chemical modifications by the TiO_2_/Ag, ZnO/Ag and TiO_2_ + ZnO/Ag composites were examined by SEM, EDS, FT-IR and XRD analyses. The SEM images presented in Figure 1 show that the untreated cotton fibres had a typical ribbon-like structure with slightly indicated bends. The application of TiO_2_/Ag, ZnO/Ag and TiO_2_ + ZnO/Ag composites significantly increased the roughness of the fibre surface. It can be clearly seen that particles of different sizes and their agglomerates were present in all three samples, i.e., CO/TiO_2_/Ag, CO/ZnO/Ag and CO/TiO_2_ + ZnO/Ag. The successful production of TiO_2_ + ZnO/Ag composite coating on the surface of the modified CO/TiO_2_ + ZnO/Ag sample was also demonstrated by the EDS analysis (Figure 1f).

**Figure 2:**
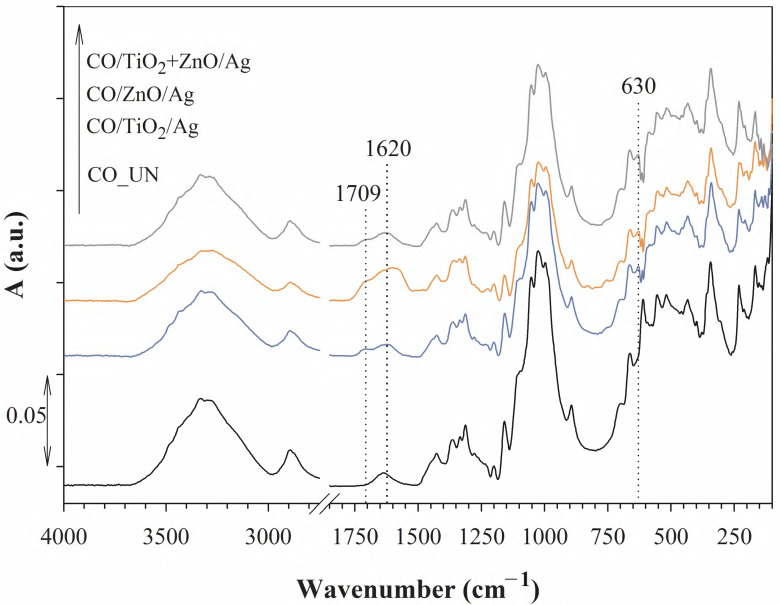
IR ATR spectra of untreated (CO_UN) and chemically modified CO/TiO_2_/Ag, CO/ZnO/Ag and CO/TiO_2_ + ZnO/Ag samples.

To investigate the influence of the chemical modification of the cotton fabric on the chemical structure of the cotton cellulose, the IR ATR spectra of the CO/TiO_2_/Ag, CO/ZnO/Ag and CO/TiO_2_ + ZnO/Ag samples (Figure 2) were analysed and compared with the IR ATR spectrum of the untreated CO_UN sample. All the investigated IR ATR spectra show characteristic absorption bands in the spectral range of 1500–800 cm^−1^, which can be attributed to the fingerprint of cellulose [47]. The main difference after chemical modification of the cotton samples is the appearance of new absorption bands at 1709 and 1620 cm^−1^, the latter having the highest intensity in the IR ATR spectrum of the CO/ZnO/Ag sample, which was blue-shifted to 1580 cm^−1^. The above absorption bands can be ascribed to the carbonyl group [47] of gallic acid, the predominant compound in the sumac leaf extract [41], which remained on the surface of cotton fibres after the in-situ synthesis of Ag NP. This is also confirmed by the appearance of a new high-intensity absorption band at 630 cm^−1^ due to the C-O-H vibration of the aromatic ring of phenol [47]. The presence of absorption bands characteristic of TiO_2_, ZnO or Ag was not detected in the IR ATR spectra of the modified CO/TiO_2_/Ag, CO/ZnO/Ag and CO/TiO_2_ + ZnO/Ag samples, indicating the absence of chemical interactions of these particles with the functional groups of cotton cellulose [47]. 

**Figure 3:**
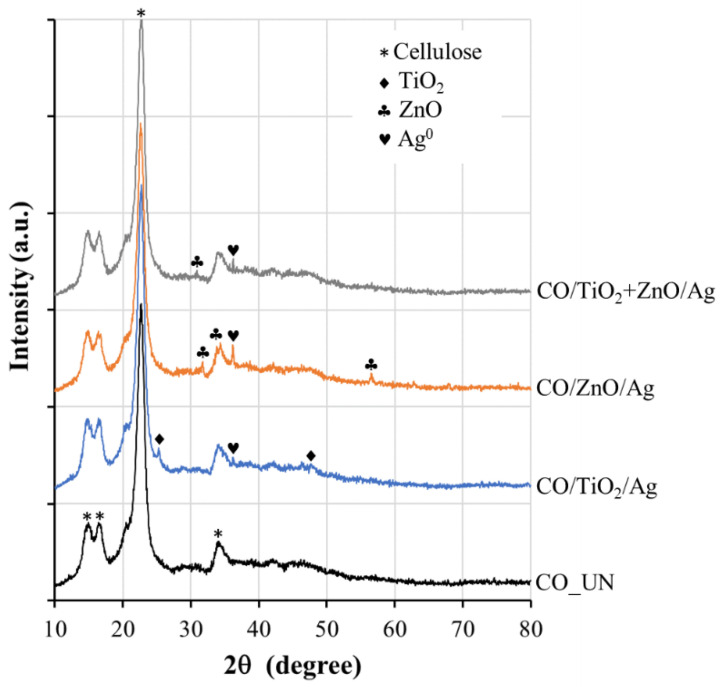
XRD patterns of untreated (CO_UN) and chemically modified CO/TiO_2_/Ag, CO/ZnO/Ag and CO/TiO_2_ + ZnO/Ag samples.

The crystallinity and crystal phases of the components in the composite coatings were exploited by the XRD analysis, and the XRD patterns are shown in Figure 3. All studied samples showed the diffraction peaks at 2θ = 15.0°, 16.8°, 22.7° and 34.5°, corresponding to the (1 1 0), (1 $\bar{1}$ 0), (2 0 0) and (4 0 0) crystallographic planes of the crystalline structure of cellulose, respectively [48]. The characteristic diffraction peaks at 2θ = 25.3° and 47.8°, corresponding to the (1 0 1) and (2 0 0) crystal planes of anatase TiO_2_, respectively, can be seen in the CO/TiO_2_/Ag sample [49]. The characteristic diffraction peaks at 2θ = 31.6°, 34.5° and 56.7°, corresponding to the (0 1 0), (0 0 2) and (1 1 0) planes of the ZnO wurtzite hexagonal phase, respectively, can be seen in the CO/ZnO/Ag sample [50]. These peaks are less visible in the CO/TiO_2_ + ZnO/Ag sample. In all the samples containing Ag, the diffraction peak at 2θ = 38.2°, corresponding to the (1 1 1), crystallographic planes of face-centred cubic silver crystals [41], is clearly visible, confirming the in-situ synthesis of Ag NPs in the CO/TiO_2_/Ag, CO/ZnO/Ag and CO/TiO_2_ + ZnO/Ag samples. These results are in agreement with the results of our previous studies [41]. 

The concentrations of TiO_2_, ZnO and Ag in the studied samples determined by ICP-MS are presented in Table 1. The results show that the loading of ZnO on the CO/ZnO sample was higher than that of TiO_2_ on the CO/TiO_2_ sample at the same sol concentration, indicating that ZnO particles are better adsorbed by cotton fibres than TiO_2_ particles. The same phenomenon was observed when the two-component TiO_2_ + ZnO sol was applied to the CO/TiO_2_ + ZnO sample. The reason for the lower adsorption of TiO_2_ compared to ZnO could be due to the agglomeration of TiO_2_ particles in the sol, which caused a lower adsorption capacity compared to the highly dispersed ZnO particles. It is also evident from Table 1 that, during the in-situ synthesis of Ag NPs, a certain amount of both TiO_2_ and ZnO was desorbed from the cotton samples, and this phenomenon was more evident for TiO_2_ than for ZnO. The same behaviour occurred for the CO/TiO_2_ + ZnO/Ag sample. Moreover, the presence of TiO_2_, ZnO and TiO_2_ + ZnO composite on the cellulose fibres increased the concentration of Ag NPs synthesized in situ, suggesting that Ag NPs are also formed on the surface of semiconductors. Table 1 also shows that Ag loading was much higher in the presence of ZnO than in the presence of TiO_2_. 

**Figure 4:**
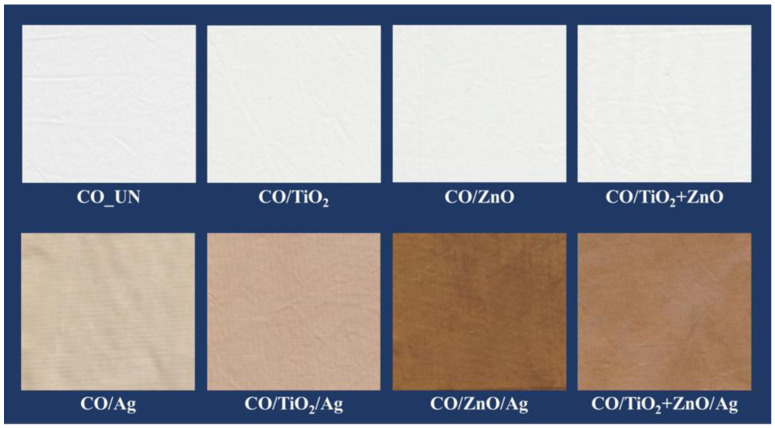
Photo images of untreated and chemically modified cotton samples.

It is also important to note that the biosynthesis of Ag particles carried out in the reduction reaction of AgNO_3_ in the presence of phenolic compounds from sumac leaf extract as reducing agents was accompanied by a colour change of the chemically modified cotton fibres to yellow–brown (Figure 4). The colour change was attributed to the surface plasmon resonance of Ag NPs, which was investigated in detail in our previous study [41].

**Figure 5:**
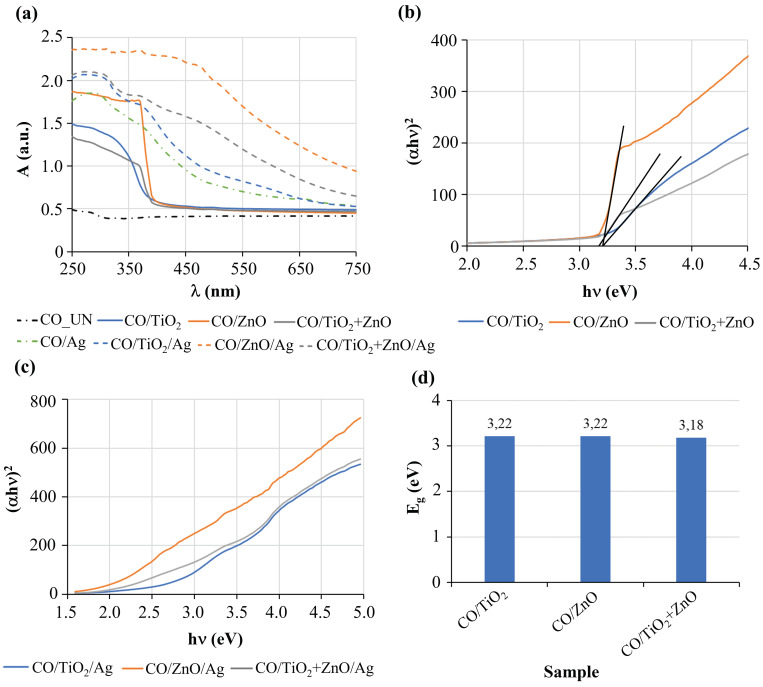
Absorption spectra of the untreated and chemically modified cotton samples (**a**); Tauc plots of the CO/TiO_2_, CO/ZnO and CO/TiO_2_ + ZnO samples (**b**); Tauc plots of the CO/TiO_2_/Ag, CO/ZnO/Ag and CO/TiO_2_ + ZnO/Ag samples (**c**); E_g_ values of chemically modified cotton samples determined from Tauc plots (**d**).

The influence of the formation of the TiO_2_ + ZnO heterostructure and the application of Ag as a co-catalyst on the optical properties of the prepared composites was investigated using the UV–Vis absorption spectra of the chemically modified cotton samples (Figure 5a). The comparison of the absorption spectra shows that the presence of TiO_2_, ZnO and TiO_2_ + ZnO coatings significantly increased the absorption of UV radiation compared to the untreated cotton. It is also evident that the absorption of the CO/ZnO sample was much higher than that of the CO/TiO_2_ sample through the whole UV range. The reason for the higher UV absorption of the CO/ZnO sample could be due to the higher ZnO concentration on the cotton fibres compared to the CO/TiO_2_ sample, but, at the same time, it also suggests that the ZnO particles are more efficient as UV absorbers compared to the TiO_2_ particles. The presence of a TiO_2_ + ZnO composite resulted in lower UV absorption of the CO/TiO_2_ + ZnO sample compared to the CO/ZnO and CO/TiO_2_ samples, although the concentration of both particles together was even higher than that of ZnO alone. The only exception was the short wavelength range of 355–390, where the absorption of the TiO_2_ + ZnO composite exceeded that of the TiO_2_ particles. This proves both the higher efficiency of ZnO as a UV absorber compared to TiO_2_ and the non-compatibility of the particles in the composite. The colouring of the cotton fibres during the in-situ biosynthesis of the Ag particles resulted in an increase in the absorbance of the CO/TiO_2_/Ag, CO/ZnO/Ag and CO/TiO_2_ + ZnO/Ag samples in the UV and visible ranges compared to the samples without Ag. The highest absorbance of a CO/ZnO/Ag sample was due to the highest Ag concentration, which was accompanied by the darkest shade (Figure 4).

From the absorption spectra, the optical band gab energies, E_g_, of the TiO_2_, ZnO and TiO_2_ + ZnO coatings on the cotton samples were determined using the Tauc relation (Figure 5b,c), in which the energy-dependent absorption coefficient, α, is related to the incident photon energy, h·ν [51,52]. This relationship is expressed by the following equation [52]:

(5) $${\left(\alpha h\right)}^{n}=K\left(h\nu -E_{g}\right),\;$$

where *K* is the absorption constant, *h* is Planck constant, ν is the frequency of light and n is an index characterizing the optical absorption process. The latter is equal to two for direct band gap transitions proposed for TiO_2_ and ZnO [52]. According to the Tauc method, the value of E_g_ was determined graphically from the Tauc plot as the value of photon energy obtained when the linear part of the plot is extrapolated to α = 0 (Figure 5b). It should be emphasized that the Tauc method can be directly applied only to semiconductor materials that do not absorb light with energy below the band gap, as is the case for TiO_2_, ZnO and TiO_2_ + ZnO (Figure 5b) [52]. In this case, the estimated values for E_g_ were 3.22 for TiO_2_ and ZnO and slightly lower, 3.18, for the TiO_2_ + ZnO composite (Figure 5d). The slight bathochromic shift in the absorption of the TiO_2_ + ZnO composite was too small to allow excitation by visible light. On the other hand, it is evident from Figure 5c that the plasmon resonance of Ag in Ag/TiO_2_, Ag/ZnO and Ag/TiO_2_ + ZnO caused by visible light absorption led to the introduction of the intra-band gap states, which showed up as a broad absorption band at energies below E_g_ (Figure 5c) [53]. In these cases, the Tauc method could not be applied because the estimated E_g_ values would be too low, and, therefore, incorrect. However, these results prove beyond doubt that visible light absorption by Ag induces the appearance of additional electron states that could affect the photocatalytic activity of the semiconductor/Ag composites.

### 3.2. Functional Properties

#### 3.2.1. Antibacterial Properties

**Figure 6:**
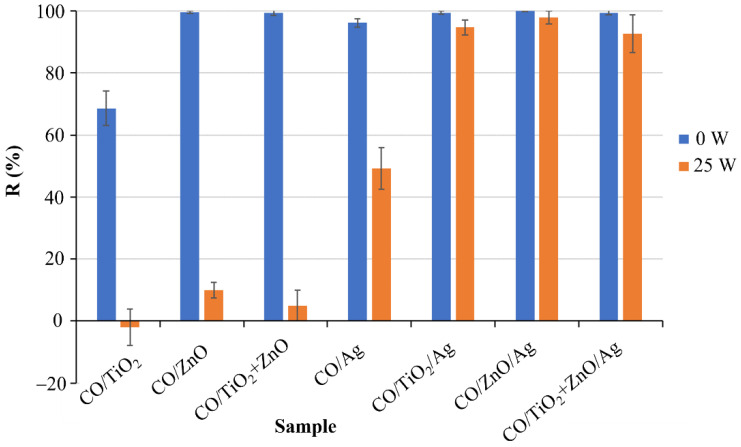
Bacterial reduction, R, of chemically modified cotton samples against *E. coli* before (0 W) and after 25 (25 W) washings.

To investigate the antibacterial activity of the chemically modified cotton samples, the reduction in growth of Gram-negative *E. coli* was determined compared to the untreated sample before and after repeated washing (Figure 6). The Gram-negative bacteria *E. coli* were selected for testing because previous studies have shown that Gram-negative bacteria are generally more resistant to the effects of TiO_2_, ZnO and Ag than Gram-positive bacteria [3,4,41]. Therefore, it was assumed that an effective antibacterial effect against *E. coli* would represent an equally good or even better effect against *S. aureus*, which is usually selected as a representative Gram-positive bacterium. The results in Figure 4 show that the photocatalytic antibacterial activity of the one-component ZnO against *E. coli* was much higher than that of the one-component TiO_2_ in the case of the unwashed (0 W) samples. The reason could be the higher concentration of ZnO than TiO_2_ on the cotton fibres or the particle structure, which was not the objective of this study. The bacterial reduction of more than 99% was also achieved by the CO/TiO_2_ + ZnO sample, proving high antibacterial activity of the composite. However, the antibacterial activity of all CO/TiO_2_, CO/ZnO and CO/TiO_2_ + ZnO samples practically disappeared after 25 washes, indicating very low wash fastness of these coatings. The Ag concentration in the one-component coating was high enough to significantly increase the antibacterial activity of the CO/Ag sample, as reflected by a 96% reduction in bacteria. As with the TiO_2_, ZnO and TiO_2_ + ZnO coatings, the wash fastness of the coating with the one-component Ag was insufficient, achieving only 50% bacterial reduction after 25 washes.

In contrast, a dramatic increase in wash fastness was obtained for the coatings in which the Ag particles were in situ biosynthesised on the cotton fibres in the presence of TiO_2_, ZnO or TiO_2_ + ZnO. Accordingly, the bacterial reductions of the CO/TiO_2_/Ag, CO/ZnO/Ag and CO/TiO_2_ + ZnO/Ag remained 94.7%, 97.9% and 93.5%, respectively, even after 25 washes (Figure 6). At first glance, this phenomenon could be explained by the higher Ag content of these samples compared to that of the CO/Ag sample (Table 2) as it was found that the increased Ag concentration in the sample led to increased antibacterial activity of the sample after washing [41]. However, a detailed comparison of our results with those of our previous research shows that, in the case of the cotton sample coated with 3500 mg/kg one-component Ag NPs, only a 65% bacterial reduction of *E. coli* was achieved after 25 washes [41]. Accordingly, it should be emphasised that the 3500 mg/kg Ag NPs had a 4.9–7.7-fold higher concentration than the concentrations determined for the CO/TiO_2_/Ag, CO/ZnO/Ag and CO/TiO_2_ + ZnO/Ag samples, which were 450, 710 and 640 mg/kg, respectively. These results clearly show that the presence of Ag in the TiO_2_/Ag, ZnO/Ag and TiO_2_ + ZnO/Ag composite coatings significantly improved the antibacterial durability of the composites.

The significant improvement in the antibacterial durability of the TiO_2_/Ag, ZnO/Ag and TiO_2_ + ZnO/Ag composite coatings compared to the TiO_2_, ZnO and TiO_2_ + ZnO coatings could be explained as follows. Despite the fact that the antimicrobial mechanism of controlled release of Ag particles requires leaching of Ag^+^ and Ag particles from the fibre surface, the content of the coatings on the CO/TiO_2_/Ag, CO/ZnO/Ag and CO/TiO_2_ + ZnO/Ag samples was still high enough to provide effective antibacterial activity even after repeated washing. This was not the case for either the coatings without Ag or the one-component Ag coating. The reason for this could be the dual antibacterial activity of the particles in the composites. Namely, the photocatalytic antibacterial activity of TiO_2_ and ZnO is directly related to their photocatalytic efficiency in generating ROS via redox reactions at the crystal surface under light irradiation. It is believed that, when TiO_2_ and ZnO come into contact with a microorganism, the photo-generated ROS can enter the cell through a diffusion process and cause oxidative destruction of all vital microbial functions [3]. In addition to the antimicrobial mechanism of controlled release of Ag^+^, electrons in the Ag surface layer excited by visible light due to the surface plasmon resonance phenomenon could importantly contribute to the generation of photocatalytic antibacterial ROS in TiO_2_ and ZnO through one of three proposed mechanisms, including photon scattering, plasmon resonance energy transfer or hot electron transfer [54].

#### 3.2.2. UV Protection Properties 

**Figure 7:**
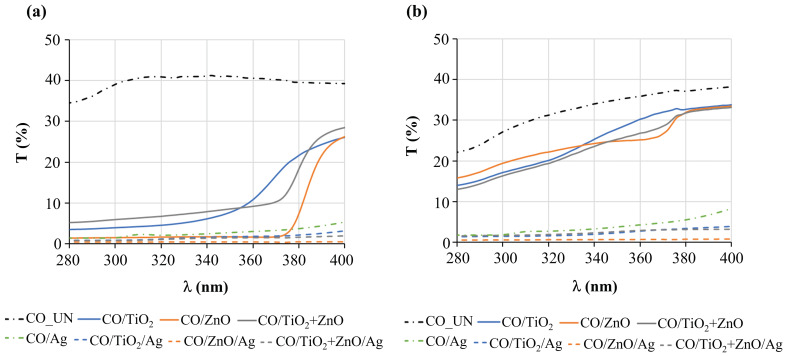
UV transmission spectra of untreated and chemically modified cotton samples before (**a**) and after 25 washings (**b**).

The UV blocking abilities of the untreated and chemically modified cotton samples before and after repeated washing were investigated using the transmission spectra in the range of 280–400 nm (Figure 7 and Table 2). 

The results show that the unwashed CO_UN sample allowed more than 30% of UV radiation through (Figure 7a), resulting in insufficient UV protection (Table 2). The presence of TiO_2_, ZnO and TiO_2_ + ZnO coatings significantly decreased the UV transmittance, and the T values decreased more in the UVB range (280–315 nm) than in the UVA range (315–400 nm) (Figure 7a). This indicates that both semiconductors are better UVB than UVA blockers. It is also clear from the results that the one-component ZnO coating provided much better UV protection than the one-component TiO_2_ and the TiO_2_ + ZnO composite coatings, indicating that these particles are incompatible in the composite. In addition, all three coatings exhibited low wash fastness, resulting in insufficient UV protection after repeated washing (Figure 7b and Table 2).

The colouration of the cotton fibres during the in situ biosynthesis of the Ag particles resulted in a drastic decrease in the UV transmission of the CO/TiO_2_/Ag, CO/ZnO/Ag and CO/TiO_2_ + ZnO/Ag samples in the UVA range (Figure 5a), which was reflected in the extremely high UVP values and the effective UVA and UVB transmittance of less than 2.5%. Thus, the highest possible protection was achieved, and all three samples were classified as “excellent anti-UVA and anti-UVB textile products” (Table 2). The UV blocking properties of the samples were only slightly affected after the 25 repeated washes, providing excellent permanent protection against UV radiation.

The results of the UV blocking properties provided the possibility to calculate the possible synergistic behaviour of Ag and semiconductors in the composite. Considering that synergy refers to the interaction of components leading to a greater effect than the sum of the individual effects, the synergistic effect, SE, could be expressed mathematically as follows [55]:

(6) $$\mathrm{SE}=\frac{{\Delta \mathrm{UPF}}_{\mathrm{observed}}}{{\Delta \mathrm{UPF}}_{\mathrm{expected}}}$$

where ΔUPF represents the difference in mean UPF value between the chemically modified sample and the untreated sample, ΔUPF_observed_ is the experimental value obtained for the composite coatings and ΔUPF_expected_ is the calculated value for the same composite with an additive effect without the synergy. The synergistic behaviour was confirmed by an SE value greater than 1. 

To correctly calculate the SE value, the concentration of each component in the composite coating should be the same as in the single-component coating. However, it can be seen in Table 2 that the Ag concentration in the one-component coating (CO/Ag sample) was lower than in the composite coatings (CO/TiO_2_/Ag, CO/ZnO/Ag and CO/TiO_2_ + ZnO/Ag samples) even though the same concentration of AgNO_3_ precursor was used for the in situ biosynthesis of Ag particles. Since the UPF value depends directly on the coating concentration, this would lead to an incorrect (too low) value for the ΔUPF_expected._ Therefore, as the best approximations, the mean UPF values of 69.1 (unwashed sample) and 35.2 (sample washed 25 times) were obtained for the CO/Ag sample, which was observed for the CO sample coated with 3500 mg/kg Ag (the CO/Ag5.0 sample) in our previous research [41]. Namely, this sample was treated with a higher AgNO_3_ concentration of 5.0 mM but under the same conditions as the CO/Ag sample in this study. The calculated SE values are summarised in Table 3 and Table 4.

From Table 3 and Table 4, it can be seen that synergistic behaviour of the components in the composite coatings was achieved for all three of CO/TiO_2_/Ag, CO/ZnO/Ag and CO/TiO_2_ + ZnO/Ag, and that the synergism between ZnO and Ag was more pronounced than that between TiO_2_ and Ag. Moreover, a significant improvement in the wash fastness of the Ag-containing composite coatings compared to the coatings without Ag was evident in increases in the SE values of the samples washed 25 times compared to the unwashed samples. This once again confirms the importance of the presence of Ag NPs for the wash durability of the coatings.

In contrast to the synergistic effect of the TiO_2_/Ag, ZnO/Ag and TiO_2_ + ZnO/Ag composites, an antagonistic effect of the TiO_2_ and ZnO components was found for the TiO_2_ + ZnO composite. Indeed, SE values equal to 0.2 and 0.6, i.e., less than 1, were calculated for the unwashed and 25-times-washed CO/TiO_2_ + ZnO samples.

#### 3.2.3. Photocatalytic Activity

**Figure 8:**
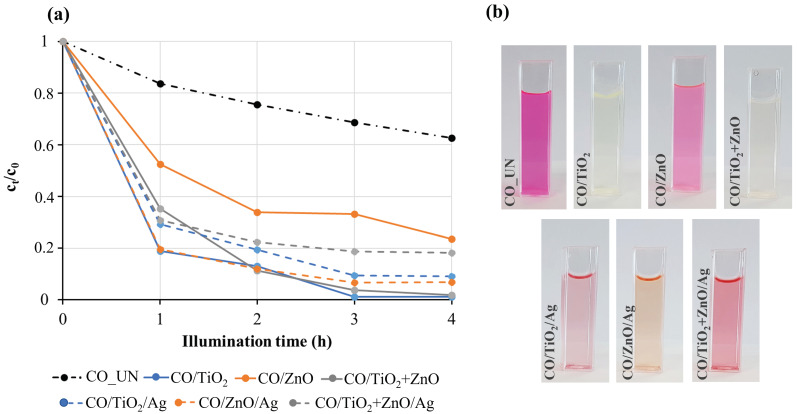
Photocatalytic degradation of Rh B in the presence of the untreated and chemically modified samples after a different illumination time (**a**); digital images of the cuvettes filled with Rh B solution after 4 h of illumination (**b**).

The results of the photocatalytic degradation of Rh B in the presence of the untreated and chemically modified samples after a different illumination time are shown in Figure 8. The results clearly show that all the chemically modified cotton samples exhibited much higher photocatalytic activity compared to the untreated cotton, resulting in higher degradation of Rh B, which was expressed as a lower c_t_/c_0_ ratio. In general, the degradation of the Rh B increased as the illumination time increased. 

Figure 8 also shows that the one component TiO_2_ coating provided the highest photodegradation efficiency for the CO/TiO_2_ sample and that the presence of ZnO in the composite coating did not improve the photocatalytic performance of the CO/TiO_2_ + ZnO sample, which remained approximately the same as that of the CO/TiO_2_ sample after four hours of illumination (Figure 8b). Moreover, the photocatalytic activity of ZnO was much lower than that of TiO_2_ despite its higher loading on cotton fibres, resulting in the lowest level of Rh B degradation in the presence of the CO/ZnO sample. However, for the CO/ZnO/Ag sample, the photocatalytic performance was significantly enhanced when the Ag NPs were synthesised in situ on the surface of the ZnO. In contrast, the presence of Ag in the TiO_2_/Ag composite did not improve the photocatalytic efficiency of the CO/TiO_2_/Ag sample compared to the CO/TiO_2_ sample but decreased it. The same was true for the CO/TiO_2_ + ZnO/Ag sample. The results clearly show that all the Ag-containing samples exhibited excellent photocatalytic activity after one hour of illumination, while their efficiency decreased with longer exposure times. The same phenomenon was also obtained by other research [56]. This suggests that the composition of these composite coatings should be optimised regarding the component concentrations. 

**Figure 9:**
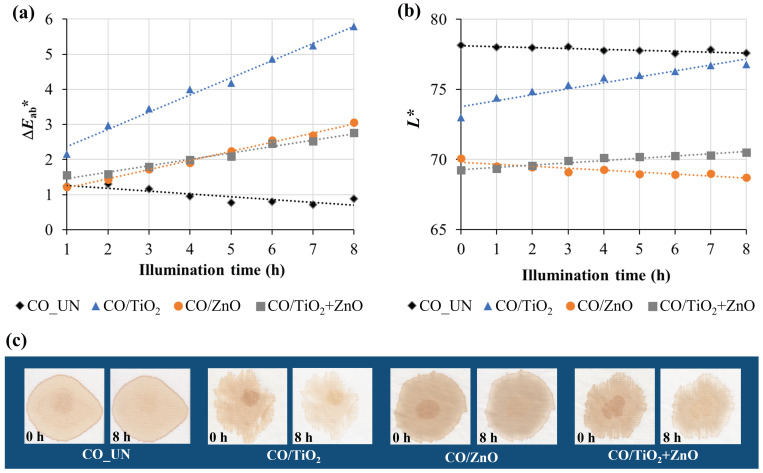
Colour difference, ΔE_ab_*, between the unilluminated samples stained by coffee and the stained samples illuminated by the simulated solar light for different periods of time (**a**); the lightness, L_ab_*, of the stained samples before and after visible light illumination for different periods of time (**b**); digital images of the samples stained by coffee before and after 8 h of visible light illumination (**c**).

The results of the photocatalytic self-cleaning performance of the untreated and chemically modified CO/TiO_2_, CO/ZnO and CO/TiO_2_ + ZnO samples are presented in Figure 9. Since the biosynthesis of Ag particles was accompanied by a yellow–brown colouration of the samples, which was more intense than the colour yield of the coffee stains, the degradation rates of the coffee stains in the CO/TiO_2_/Ag, CO/ZnO/Ag and CO/TiO_2_ + ZnO/Ag samples could not be quantitatively evaluated. 

As shown in Figure 9a,b, the values of ΔE_ab_* and L* were the criteria for photocatalytic self-cleaning efficiency of the samples. The results show that, for the CO_UN sample, the values of ΔE_ab_* and L* decreased after illumination, indicating that the untreated cotton could not degrade the coffee stain. The colour of the stain even darkened when exposed to the light source. On the other hand, the presence of TiO_2_, ZnO and TiO_2_ + ZnO coatings on the cotton samples resulted in increased ΔE_ab_* values after illumination. From the comparison of the ΔE_ab_* and L* values, it can be seen that the degradation rate of the coffee stain was significantly higher for the CO/TiO_2_ sample than for the CO/ZnO and CO/TiO_2_ + ZnO samples, which can also be clearly seen in the digital images (Figure 9c). The high ΔE_ab_* value was due to the strong fading of the coffee stain on the CO/TiO_2_ sample, which manifested itself in an increase in colour lightness. This phenomenon was much less pronounced on the CO/ZnO and CO/TiO_2_ + ZnO samples, suggesting their lower photocatalytic efficiency. These results are consistent with those of the photocatalytic degradation of Rh B.

**Figure 10:**
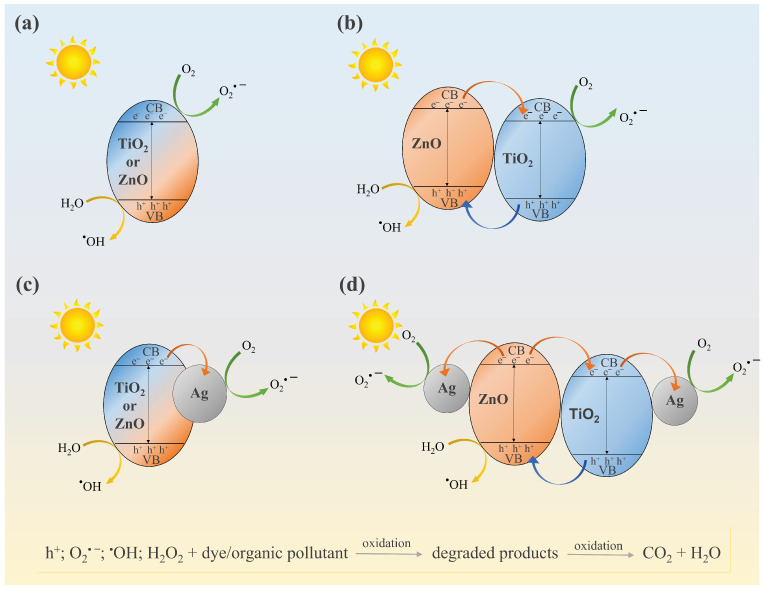
Schematic presentation of the proposed photocatalytic mechanisms of TiO_2_ or ZnO (**a**), TiO_2_ + ZnO (**b**), TiO_2_/Ag or ZnO/Ag (**c**) and TiO_2_ + ZnO/Ag (**d**) composites on the chemically modified cotton samples.

The possible mechanisms of photocatalytic degradation of dyes and organic pollutants by the one component TiO_2_ or ZnO, and TiO_2_ + ZnO, TiO_2_/Ag, ZnO/Ag and TiO_2_ + ZnO/Ag composites on the chemically modified cotton samples are schematically shown in Figure 10. The mechanism of photocatalytic activity of the one component TiO_2_ or ZnO (Figure 10a) is related to the excitation of electrons from the valence band (VB) into the conduction band (CB) under the UV light irradiation, creating holes in the VB. The photoexcited electrons in CB can react with oxygen on the crystal surface to form superoxide radicals (O_2_^•−^) in the reduction reaction. The photogenerated holes in VB can react with water on the crystal surface to form hydroxyl radicals (^•^OH) in the oxidation reaction. The formation of ROS is crucial for the degradation of dyes and organic pollutants during the photooxidation reaction. The photocatalytic efficiency could be affected by the rapid recombination of electron–hole pairs [3,4]. 

In the case of the TiO_2_ + ZnO composite, the formation of a type-II heterojunction (Figure 10b) is proposed. In this case, the photogenerated electrons from the CB of ZnO are transferred to the CB of TiO_2_, since the CB of ZnO is more negative than the CB of TiO_2_. Moreover, the holes are transferred from the VB of TiO_2_ to the VB of ZnO since the VB of TiO_2_ is more positive than the VB of ZnO. These processes lead to the separation of electrons and holes and prevent the recombination of electron–hole pairs, improving the redox process. The electrons accumulated in the CB of TiO_2_ can participate in the photoreduction reaction, and the holes accumulated in the VB of ZnO can participate in the photooxidation reaction [24,28]. 

The photocatalytic mechanism of TiO_2_/Ag and ZnO/Ag suggests the transfer of the photogenerated electrons from the semiconductor CB to Ag due to the lower Fermi energy of Ag compared to the semiconductors. This leads to the formation of a Schottky barrier, which causes Ag to trap the electrons and prevents the electron–hole recombination. The electrons trapped by Ag can participate in the photoreduction reaction, while the holes in the VC of the semiconductor can participate in the photooxidation reaction. It is believed that the presence of Ag^0^, which acts as a current collector and plasmonic absorber, significantly increases the photocatalytic efficiency of the composite [15,18,39,56,57].

The photocatalytic mechanism of the TiO_2_ + ZnO/Ag heterostructure is very complex and not thoroughly investigated. It is directly influenced by the synthesis pathway, which affects the composition of the heterostructure [37,38,40]. One of the possible mechanisms is the formation of a type-II heterojunction between TiO_2_ and ZnO with the simultaneous formation of a Schottky barrier in the contact between the semiconductors and Ag NPs (Figure 10d). To discuss the mechanism in detail, further studies should be carried out. However, the results of the photocatalytic activity of the CO/ TiO_2_ + ZnO/Ag sample suggest that the all-solid-state Z-scheme photocatalyst system, in which Ag is used as an excellent electron mediator and which exhibits excellent photocatalytic efficiency [57], did not result in the chemical modification of cotton fibres. 

## 4. Conclusions

In summary, this study presented a novel, environmentally friendly and facile method for producing durable antimicrobial and UV blocking properties in cotton fibres. The method consists of the application of TiO_2_, ZnO and TiO_2_ + ZnO particles to cotton fibres, followed by in situ biosynthesis of Ag NPs in the presence of sumac leaf extract as a reducing agent.

The results showed that the loading of ZnO particles was higher than that of TiO_2_ particles despite the same concentrations of precursors and that the presence of TiO_2_ increased the loading of ZnO in the two-component TiO_2_ + ZnO coating. Moreover, the presence of TiO_2_, ZnO and TiO_2_ + ZnO coatings increased the concentration of Ag particles synthesised in-situ, and the Ag concentration in the coatings increased as follows: Ag < TiO_2_/Ag < TiO_2_ + ZnO/Ag < ZnO/Ag. The formation of Ag NPs was accompanied by a colour change of the chemically modified cotton fibres to yellow–brown; the highest Ag concentration was associated with the darkest CO/ZnO/Ag sample.

The in-situ synthesis of Ag NPs on the TiO_2_, ZnO and TiO_2_ + ZnO coatings not only increased their antimicrobial activity compared to the TiO_2_, ZnO and TiO_2_ + ZnO coatings but also dramatically increased antimicrobial durability, with a bacterial reduction of more than 93% even after 25 washes. In addition, the presence of Ag NPs dramatically reduced the UV transmission of the CO/TiO_2_/Ag, CO/ZnO/Ag and CO/TiO_2_ + ZnO/Ag samples and provided excellent protection against UV radiation, as shown by UPF values of 50+, even after 25 washes. The results of UV blocking properties showed the synergistic effects between Ag and TiO_2_ and Ag and ZnO in the composite coatings. In contrast to the coatings containing Ag NPs, an antagonistic effect of the TiO_2_ and ZnO components was observed in the TiO_2_ + ZnO coating. The antagonistic behaviour also resulted in the ineffective self-cleaning activity of the TiO_2_ + ZnO coating compared to the one-component TiO_2_ coating, although the E_g_ of the TiO_2_ + ZnO composite exhibited a slight bathochromic shift in absorption compared to TiO_2_ and ZnO. However, the as prepared TiO_2_/Ag, ZnO/Ag and TiO_2_ + ZnO/Ag coatings were characterised by their excellent and durable antimicrobial and UV protection properties.

## Figures and Tables

**Table 1:** Concentration of TiO_2_, ZnO and Ag on the studied samples.

Sample	Concentration (mg/kg)		
	TiO_2_	ZnO	Ag
CO/TiO_2_	14,680	0	0
CO/ZnO	0	16,180	0
CO/TiO_2_ + ZnO	7670	9460	0
CO/Ag	0	0	340
CO/TiO_2_/Ag	4170	0	450
CO/ZnO/Ag	0	12,430	710
CO/TiO_2_ + ZnO/Ag	1180	1320	640

**Table 2:** The arithmetic mean of T in the UVA, UVB and UVR ranges; the mean UPF; the UVR protection categories for the untreated and chemically modified cotton samples and the synergistic effect, SE, between the components in the composite coatings.

Sample	Number of Washings	T (UVA) (%)	T (UVB) (%)	T (UVR) (%)	Mean UPF Value	UVR Protection Category *
CO	0	37.8	35.5	37.2	2.7	NR
	25	35.3	27.4	33.44	3.4	NR
CO/TiO_2_	0	13.1	4.0	11.0	20.6	G
	25	28.4	17.3	25.8	5.2	NR
CO/ZnO	0	6.4	1.5	5.3	55.7	E
	25	26.9	19.6	25.2	4.7	NR
CO/TiO_2_ + ZnO	0	13.0	6.0	11.4	15.9	G
	25	26.7	16.6	24.3	5.4	NR
CO/Ag	0	3.3	1.9	2.9	47.9	E
	25	6.4	2.8	4.9	33.2	VG
CO/TiO_2_/Ag	0	1.9	0.9	1.7	92.1	E
	25	2.7	1.5	2.4	61.0	E
CO/ZnO/Ag	0	0.5	0.4	0.5	229.0	E
	25	0.7	0.6	0.7	164.9	E
CO/TiO_2_ + ZnO/Ag	0	1.5	0.8	1.3	105.4	E
	25	2.6	1.7	2.4	55.3	E

* NR—non-rateable, G—good, VG—very good, E—excellent.

**Table 3:** Calculated $\Delta UPF_{observed}$ and SE between the components in the unwashed composite coatings.

Sample	$\Delta {\mathrm{UPF}}_{\mathrm{observed}}$	$\frac{\Delta {\mathrm{UPF}}_{\mathrm{observed}}}{\Delta {\mathrm{UPF}}_{\mathrm{expected}}}$	SE
CO/Ag *	66.4	$\frac{89.4}{17.9+66.4}$	1.1
CO/TiO_2_	17.9		
CO/TiO_2_/Ag	89.4		
CO/ZnO	53.1	$\frac{226.3}{53.1+66.4}$	1.9
CO/ZnO/Ag	226.3		
CO/TiO_2_ + ZnO	13.2	$\frac{102.7}{13.2+66.4}$	1.3
CO/TiO_2_ + ZnO/Ag	102.7		

* The mean UPF value is selected to be 69.1. Adapted with permission from [41]. Copyright, 2022, Elsevier.

**Table 4:** Calculated $\Delta UPF_{observed}$ and the synergistic effect, SE, between the components in the 25-times-washed composite coatings.

Sample	$\Delta {\mathrm{UPF}}_{\mathrm{observed}}$	ΔUPFobserved ΔUPFexpected	SE
CO/Ag *	31.8	$\frac{57.9}{1.8+31.8}$	1.7
CO/TiO_2_	1.8		
CO/TiO_2_/Ag	57.9		
CO/ZnO	1.3	$\frac{161.5}{1.3+31.8}$	4.9
CO/ZnO/Ag	161.5		
CO/TiO_2_ + ZnO	2.0	$\frac{51.9}{2.0+31.8}$	1.5
CO/TiO_2_ + ZnO/Ag	51.9		

* The mean UPF value is selected to be 35.2. Adapted with permission from [41]. Copyright, 2022, Elsevier.

## Data Availability

Not applicable.
